# Redetermination of Zn_2_Mo_3_O_8_

**DOI:** 10.1107/S1600536809021928

**Published:** 2009-06-13

**Authors:** Jerome Cuny, Patrick Gougeon, Philippe Gall

**Affiliations:** a Sciences Chimiques de Rennes, UMR CNRS No. 6226, Université de Rennes I–INSA Rennes, Avenue du Général Leclerc, 35042 Rennes CEDEX, France

## Abstract

The crystal structure of dizinc trimolybdenum(IV) octa­oxide, Zn_2_Mo_3_O_8_, has been redetermined from single-crystal X-ray data. The structure has been reported previously based on neutron powder diffraction data [Hibble *et al.* (1999 ▶). *Acta Cryst.* B**55**, 683-697] and single-crystal data [McCarroll *et al.* (1957 ▶). *J. Am. Chem. Soc.* **79**, 5410–5414; Ansell & Katz (1966 ▶) *Acta Cryst.* **21**, 482–485]. The results of the current redetermination show an improvement in the precision of the structural and geometric parameters with all atoms refined with anisotropic displacement parameters. The crystal structure consists of distorted hexa­gonal-close-packed oxygen layers with stacking sequence *abac* along [001] and is held together by alternating zinc and molybdenum layers. The Zn atoms occupy both tetra­hedral and octa­hedral inter­stices with a ratio of 1:1. The Mo atoms occupy octa­hedral sites and form strongly bonded triangular clusters involving three MoO_6_ octa­hedra that are each shared along two edges, forming a Mo_3_O_13_ unit. All atoms lie on special positions. The Zn atoms are in 2*b* Wyckoff positions with 3*m*. site symmetry, the Mo atoms are in 6*c* Wyckoff positions with . *m*. site symmetry and the O atoms are in 2*a*, 2*b* and 6*c* Wyckoff positions with 3*m*. and . *m*. site symmetries, respectively.

## Related literature

The synthesis of Zn_2_Mo_3_O_8_ is described by McCarroll *et al.* (1957 ▶). For previous reports of the crystal structure, see: McCarroll *et al.* (1957 ▶); Ansell & Katz (1966 ▶); Hibble *et al.* (1999 ▶). Zn_2_Mo_3_O_8_ is isotypic with the mineral kamiokite, Fe_2_Mo_3_O_8_ (Kanazawa & Sasaki, 1986 ▶). For other compounds containing Mo_3_O_13_ cluster units, see: Betteridge *et al.* (1984 ▶); Collins *et al.* (1989 ▶); Gall & Gougeon (2005 ▶); Gougeon & Gall (2007 ▶); McCarroll (1977 ▶); Torardi & McCarley (1985 ▶).

## Experimental

### 

#### Crystal data

Zn_2_Mo_3_O_8_
*M* *_r_* = 546.56
Hexagonal, 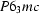

*a* = 5.7816 (2) Å
*c* = 9.9345 (3) Å
*V* = 287.59 (2) Å^3^
*Z* = 2
Mo *K*α radiation
μ = 14.59 mm^−1^
*T* = 293 K
0.21 × 0.13 × 0.07 mm

#### Data collection

Nonius KappaCCD diffractometer
Absorption correction: analytical (de Meulenaer & Tompa, 1965 ▶) *T*_min_ = 0.048, *T*_max_ = 0.157
8536 measured reflections
790 independent reflections
778 reflections with *I* > 2σ(*I*)
*R*_int_ = 0.023

#### Refinement

*R*[*F* ^2^ > 2σ(*F* ^2^)] = 0.013
*wR*(*F* ^2^) = 0.029
*S* = 1.16
790 reflections
33 parameters
1 restraint
Δρ_max_ = 0.97 e Å^−3^
Δρ_min_ = −1.06 e Å^−3^
Absolute structure: Flack (1983 ▶), 322 Friedel pairs
Flack parameter: 0.155 (15)

### 

Data collection: *COLLECT* (Nonius, 1998 ▶); cell refinement: *COLLECT*; data reduction: *EVALCCD* (Duisenberg *et al.*, 2003 ▶); program(s) used to solve structure: *SIR97* (Altomare *et al.*, 1999 ▶); program(s) used to refine structure: *SHELXL97* (Sheldrick, 2008 ▶); molecular graphics: *DIAMOND* (Bergerhoff, 1996 ▶); software used to prepare material for publication: *SHELXL97*.

## Supplementary Material

Crystal structure: contains datablocks I, global. DOI: 10.1107/S1600536809021928/wm2235sup1.cif

Structure factors: contains datablocks I. DOI: 10.1107/S1600536809021928/wm2235Isup2.hkl

Additional supplementary materials:  crystallographic information; 3D view; checkCIF report

## Figures and Tables

**Table 1:** Selected bond lengths (Å)

Mo1—O1	1.9549 (14)
Mo1—O4	2.0286 (19)
Mo1—O2	2.0804 (10)
Mo1—O3	2.1415 (14)
Mo1—Mo1^i^	2.5326 (2)
Zn1—O3^ii^	1.963 (3)
Zn2—O1	2.0467 (18)
Zn2—O2^iii^	2.1431 (16)

Symmetry codes: (i) 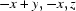; (ii) 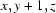; (iii) 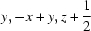.

**Fig. 1.**
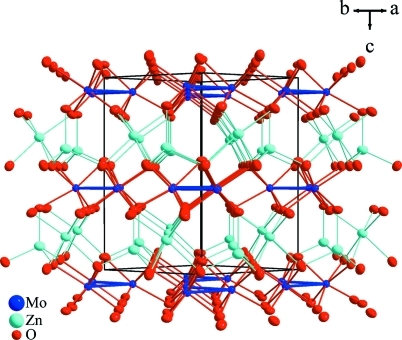
: View of Zn2Mo3O8 along [110].

**Fig. 2.**
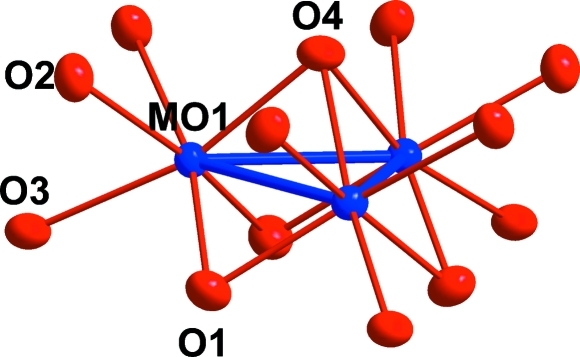
: Plot showing the atom-numbering scheme of the Mo3O13 cluster unit. Displacement ellipsoids are drawn at the 97% probability level.

## supplementary crystallographic information

### Comment

The *M*_2_Mo_3_O_8_ compounds, where *M* is a divalent metal such as Mg, Zn, Fe, Co, Ni, Zn and Cd, were first synthesized by McCarroll *et al.* (1957). They presented the results of structure determination on Zn_2_Mo_3_O_8_ from photographic data (*R* = 0.118). Later, a refinement of the structure was accomplished by Ansell & Katz (1966) with a *R* factor of 0.069. Among the above compounds, it is interesting to note that Fe_2_Mo_3_O_8_ is a mineral known as kamiokite (Kanazawa & Sasaki, 1986). The main structural feature of Zn_2_Mo_3_O_8_ is the occurrence of Mo_3_O_13_ cluster units sharing part of their oxygen atoms to form layers according to the connective formula Mo_3_O_4_O_6/2_O_3/3_. The oxygen atoms form an hexagonal-close-packing with a stacking sequence *abac* along [001] (Fig. 1). The Mo—Mo distance within the Mo_3_ triangle (Fig. 2) is 2.5326 (2) Å which differs slightly from 2.524 (2) Å found previously and is equal to that found in the isotopic compound Fe_2_Mo_3_O_8_ (2.5326 (5) Å). The Mo—O distances range from 1.9549 (14) to 2.1415 (14) Å compared to 1.928 (20) to 2.128 (30) Å in the previous determination based on single-crystal data (1.953 (4)–2.135 (4) in Fe_2_Mo_3_O_8_). In our work, The ZnO_4_ tetrahedra appear more regular with Zn—O distances of 1.963 (3) and 1.9687 (15) Å instead of 1.98 (1) and 1.99 (3) Å while the ZnO_6_ octahedra are more distorted with Zn—O distances of 2.0467 (18) and 2.1431 (16) Å compared to 2.072 (20) and 2.123 (10) Å observed by Ansell & Katz (1966).

For other compounds containing Mo_3_O_13_ cluster units, see: Betteridge *et al.* (1984); Collins *et al.* (1989); Gall & Gougeon (2005); Gougeon & Gall (2007); McCarroll (1977); Torardi & McCarley (1985).

### Experimental

Single crystals of Zn_2_Mo_3_O_8_ were obtained by the reaction of ZnO, MoO_3_, and Mo. The initial mixture (*ca* 5 g) was cold pressed and loaded into a molybdenum crucible, which was sealed under a low argon pressure using an arc welding system. The charge was heated at the rate of 300 K/h up to 1573 K, the temperature which was held for 48 h, then cooled at 100 K/h down to 1373 K and finally cooled down to room temperature by switching off the furnace.

### Refinement

The highest peak and the deepest hole are located 0.68 Å and 0.74 Å from Mo1. The crystal under investigation was racemically twinned with a twin component ratio of 0.155 (15):0.845 (155).

### Crystal data

**Table:**

Zn_2_Mo_3_O_8_	*D*_x_ = 6.312 Mg m^−^^3^
*M**_r_* = 546.56	Mo *K*α radiation, λ = 0.71069 Å
Hexagonal, *P*6_3_*m**c*	Cell parameters from 6245 reflections
Hall symbol: P 6c -2c	θ = 0.9–44.0°
*a* = 5.7816 (2) Å	µ = 14.59 mm^−^^1^
*c* = 9.9345 (3) Å	*T* = 293 K
*V* = 287.59 (2) Å^3^	Irregular block, black
*Z* = 2	0.21 × 0.13 × 0.07 mm
*F*(000) = 500	

### Data collection

**Table:**

Nonius KappaCCD diffractometer	790 independent reflections
Radiation source: fine-focus sealed tube	778 reflections with *I* > 2σ(*I*)
graphite	*R*_int_ = 0.023
φ scans (κ = 0) + additional ω scans	θ_max_ = 44.0°, θ_min_ = 4.1°
Absorption correction: analytical (de Meulenaer & Tompa, 1965)	*h* = −11→7
*T*_min_ = 0.048, *T*_max_ = 0.157	*k* = −11→11
8536 measured reflections	*l* = −16→19

### Refinement

**Table:**

Refinement on *F*^2^	Secondary atom site location: difference Fourier map
Least-squares matrix: full	*w* = 1/[σ^2^(*F*_o_^2^) + (0.0117*P*)^2^ + 0.2665*P*] where *P* = (*F*_o_^2^ + 2*F*_c_^2^)/3
*R*[*F*^2^ > 2σ(*F*^2^)] = 0.013	(Δ/σ)_max_ = 0.001
*wR*(*F*^2^) = 0.029	Δρ_max_ = 0.97 e Å^−^^3^
*S* = 1.16	Δρ_min_ = −1.06 e Å^−^^3^
790 reflections	Extinction correction: *SHELXL97* (Sheldrick, 2008), Fc^*^=kFc[1+0.001xFc^2^λ^3^/sin(2θ)]^-1/4^
33 parameters	Extinction coefficient: 0.0306 (13)
1 restraint	Absolute structure: Flack (1983), 322 Friedel pairs
Primary atom site location: structure-invariant direct methods	Flack parameter: 0.155 (15)

### Special details

**Table:**

Geometry. All e.s.d.'s (except the e.s.d. in the dihedral angle between two l.s. planes) are estimated using the full covariance matrix. The cell e.s.d.'s are taken into account individually in the estimation of e.s.d.'s in distances, angles and torsion angles; correlations between e.s.d.'s in cell parameters are only used when they are defined by crystal symmetry. An approximate (isotropic) treatment of cell e.s.d.'s is used for estimating e.s.d.'s involving l.s. planes.
Refinement. Refinement of *F*^2^ against ALL reflections. The weighted *R*-factor *wR* and goodness of fit *S* are based on *F*^2^, conventional *R*-factors *R* are based on *F*, with *F* set to zero for negative *F*^2^. The threshold expression of *F*^2^ > σ(*F*^2^) is used only for calculating *R*-factors(gt) *etc*. and is not relevant to the choice of reflections for refinement. *R*-factors based on *F*^2^ are statistically about twice as large as those based on *F*, and *R*- factors based on ALL data will be even larger.

### Fractional atomic coordinates and isotropic or equivalent isotropic displacement parameters (Å^2^)

**Table:**

	*x*	*y*	*z*	*U*_iso_*/*U*_eq_	
Mo1	−0.29203 (3)	−0.146014 (13)	−0.925032 (13)	0.00373 (3)	
Zn1	−0.6667	0.6667	−0.62348 (6)	0.00671 (7)	
Zn2	−0.3333	0.3333	−0.68932 (6)	0.00606 (7)	
O1	−0.16669 (16)	0.16669 (16)	−0.8086 (2)	0.00588 (19)	
O2	−0.51182 (15)	−0.0236 (3)	−1.04041 (16)	0.0055 (2)	
O3	−0.6667	−0.3333	−0.8210 (3)	0.0054 (4)	
O4	0.0000	0.0000	−1.0666 (3)	0.0057 (3)	

### Atomic displacement parameters (Å^2^)

**Table:**

	*U*^11^	*U*^22^	*U*^33^	*U*^12^	*U*^13^	*U*^23^
Mo1	0.00333 (5)	0.00389 (4)	0.00377 (5)	0.00166 (2)	0.00017 (5)	0.00008 (2)
Zn1	0.00746 (10)	0.00746 (10)	0.00521 (15)	0.00373 (5)	0.000	0.000
Zn2	0.00647 (10)	0.00647 (10)	0.00525 (15)	0.00323 (5)	0.000	0.000
O1	0.0061 (4)	0.0061 (4)	0.0052 (5)	0.0028 (4)	0.0007 (2)	−0.0007 (2)
O2	0.0048 (3)	0.0056 (5)	0.0066 (6)	0.0028 (2)	0.0005 (2)	0.0011 (4)
O3	0.0063 (5)	0.0063 (5)	0.0038 (8)	0.0031 (2)	0.000	0.000
O4	0.0068 (5)	0.0068 (5)	0.0036 (8)	0.0034 (3)	0.000	0.000

### Geometric parameters (Å, °)

**Table:**

Mo1—O1	1.9549 (14)	Zn1—O2^v^	1.9687 (15)
Mo1—O1^i^	1.9549 (14)	Zn1—O2^vi^	1.9687 (15)
Mo1—O4	2.0286 (19)	Zn1—O2^vii^	1.9687 (15)
Mo1—O2	2.0804 (10)	Zn2—O1	2.0467 (18)
Mo1—O2^ii^	2.0804 (10)	Zn2—O1^viii^	2.0467 (18)
Mo1—O3	2.1415 (14)	Zn2—O1^ix^	2.0467 (18)
Mo1—Mo1^iii^	2.5326 (2)	Zn2—O2^x^	2.1431 (16)
Mo1—Mo1^i^	2.5326 (2)	Zn2—O2^xi^	2.1431 (16)
Zn1—O3^iv^	1.963 (3)	Zn2—O2^vii^	2.1431 (16)
			
O1—Mo1—O1^i^	95.38 (11)	O1—Zn2—O1^viii^	89.84 (8)
O1—Mo1—O4	100.30 (5)	O1—Zn2—O1^ix^	89.84 (8)
O1^i^—Mo1—O4	100.30 (5)	O1^viii^—Zn2—O1^ix^	89.84 (8)
O1—Mo1—O2	91.06 (7)	O1—Zn2—O2^x^	96.01 (5)
O1^i^—Mo1—O2	166.69 (5)	O1^viii^—Zn2—O2^x^	171.73 (8)
O4—Mo1—O2	89.96 (6)	O1^ix^—Zn2—O2^x^	96.01 (5)
O1—Mo1—O2^ii^	166.69 (5)	O1—Zn2—O2^xi^	96.01 (5)
O1^i^—Mo1—O2^ii^	91.06 (7)	O1^viii^—Zn2—O2^xi^	96.01 (5)
O4—Mo1—O2^ii^	89.96 (6)	O1^ix^—Zn2—O2^xi^	171.73 (7)
O2—Mo1—O2^ii^	80.41 (8)	O2^x^—Zn2—O2^xi^	77.60 (7)
O1—Mo1—O3	89.76 (6)	O1—Zn2—O2^vii^	171.73 (7)
O1^i^—Mo1—O3	89.76 (6)	O1^viii^—Zn2—O2^vii^	96.01 (5)
O4—Mo1—O3	164.96 (9)	O1^ix^—Zn2—O2^vii^	96.01 (5)
O2—Mo1—O3	78.60 (6)	O2^x^—Zn2—O2^vii^	77.60 (7)
O2^ii^—Mo1—O3	78.60 (6)	O2^xi^—Zn2—O2^vii^	77.60 (7)
O1—Mo1—Mo1^iii^	49.63 (4)	Mo1—O1—Mo1^iii^	80.75 (7)
O1^i^—Mo1—Mo1^iii^	95.26 (4)	Mo1—O1—Zn2	137.21 (4)
O4—Mo1—Mo1^iii^	51.38 (4)	Mo1^iii^—O1—Zn2	137.21 (4)
O2—Mo1—Mo1^iii^	97.78 (4)	Zn1^xii^—O2—Mo1	119.89 (5)
O2^ii^—Mo1—Mo1^iii^	141.34 (3)	Zn1^xii^—O2—Mo1^xiii^	119.89 (5)
O3—Mo1—Mo1^iii^	139.34 (4)	Mo1—O2—Mo1^xiii^	102.68 (7)
O1—Mo1—Mo1^i^	95.26 (4)	Zn1^xii^—O2—Zn2^xiv^	111.56 (8)
O1^i^—Mo1—Mo1^i^	49.63 (4)	Mo1—O2—Zn2^xiv^	99.62 (5)
O4—Mo1—Mo1^i^	51.38 (4)	Mo1^xiii^—O2—Zn2^xiv^	99.62 (5)
O2—Mo1—Mo1^i^	141.34 (3)	Zn1^xv^—O3—Mo1^xiii^	118.84 (7)
O2^ii^—Mo1—Mo1^i^	97.78 (4)	Zn1^xv^—O3—Mo1^ii^	118.84 (7)
O3—Mo1—Mo1^i^	139.34 (4)	Mo1^xiii^—O3—Mo1^ii^	98.68 (9)
Mo1^iii^—Mo1—Mo1^i^	60.0	Zn1^xv^—O3—Mo1	118.84 (7)
O3^iv^—Zn1—O2^v^	114.79 (5)	Mo1^xiii^—O3—Mo1	98.68 (9)
O3^iv^—Zn1—O2^vi^	114.79 (5)	Mo1^ii^—O3—Mo1	98.68 (9)
O2^v^—Zn1—O2^vi^	103.67 (6)	Mo1^iii^—O4—Mo1	77.25 (8)
O3^iv^—Zn1—O2^vii^	114.79 (5)	Mo1^iii^—O4—Mo1^i^	77.25 (8)
O2^v^—Zn1—O2^vii^	103.67 (6)	Mo1—O4—Mo1^i^	77.25 (8)
O2^vi^—Zn1—O2^vii^	103.67 (6)		

Symmetry codes: (i) −*y*, *x*−*y*, *z*; (ii) −*x*+*y*−1, −*x*−1, *z*; (iii) −*x*+*y*, −*x*, *z*; (iv) *x*, *y*+1, *z*; (v) −*x*−1, −*y*+1, *z*+1/2; (vi) *y*−1, −*x*+*y*, *z*+1/2; (vii) *x*−*y*, *x*+1, *z*+1/2; (viii) −*x*+*y*−1, −*x*, *z*; (ix) −*y*, *x*−*y*+1, *z*; (x) *y*, −*x*+*y*, *z*+1/2; (xi) −*x*−1, −*y*, *z*+1/2; (xii) −*x*−1, −*y*+1, *z*−1/2; (xiii) −*y*−1, *x*−*y*, *z*; (xiv) −*x*−1, −*y*, *z*−1/2; (xv) *x*, *y*−1, *z*.
